# LRH1 Promotes Tumor Cell Proliferation and Migration and Is Correlated With Poor Prognosis in Ovarian Cancer

**DOI:** 10.3389/fonc.2020.583566

**Published:** 2020-10-20

**Authors:** Wenzhou Sun, Qingtao Shi, Jiaxin Li, Jinmeng Li, Libo Yu

**Affiliations:** ^1^Department of Medical Oncology, Harbin Medical University Cancer Hospital, Harbin Medical University, Harbin, China; ^2^Department of Pathology, Harbin Medical University Cancer Hospital, Harbin Medical University, Harbin, China; ^3^Department of Gynecology, Harbin Medical University Cancer Hospital, Harbin Medical University, Harbin, China

**Keywords:** liver receptor homolog 1 (LRH1), ovarian cancer, proliferation, metastasis, prognosis

## Abstract

**Background:**

Liver receptor homolog 1 (LRH1) plays a vital role in several human cancers, but its role in ovarian cancer (OC) remains unclear. We aimed to explore the functions of LRH1 and its clinical relevance.

**Methods:**

LRH1 expression was evaluated by immunohistochemistry and reverse transcription quantitative polymerase chain reaction (RT-qPCR). The effects of LRH1 on tumor cell proliferation, migration and epithelial–mesenchymal transition (EMT) were evaluated *in vitro*. Furthermore, bioinformatics analysis was applied to predict the functions of LRH1.

**Results:**

RT-qPCR showed that LRH1 mRNA expression was higher in the invasive lesions (*P* < 0.05). LRH1 overexpression was extremely related with elevated International Federation of Gynecology and Obstetrics (FIGO) stage (*P* = 0.001), lymph node metastasis (*P* = 0.011), peritoneal metastasis (*P* = 0.001), and platinum resistance (*P* = 0.037). Furthermore, LRH1 expression was an independent prognostic index for disease-free survival in patients with OC (*P* = 0.041). LRH1 overexpression (*P* = 0.011), FIGO stage (*P* < 0.001), and ascites (*P* = 0.015) independently affected peritoneal metastasis in patients with OC. LRH1 knockdown significantly inhibited the proliferation, migration, and EMT of human OC cells (*P* < 0.05); however, it reversed cisplatin resistance. Bioinformatics analysis indicated that the functions of LRH1 were associated with the PRC1 complex, nuclear ubiquitin ligase complex, and Polycomb-group (PcG) proteins.

**Conclusions:**

This study provides evidence of the predictive value of LRH1 on peritoneal metastasis and poor outcome and highlights the potential role of LRH1 as a biomarker for the targeted therapy of OC. Furthermore, LRH1 promotes OC cell proliferation, migration, and EMT *in vitro*, and its functions may be associated with PRC1 complex, nuclear ubiquitin ligase complex, and PcG proteins.

## Background

Ovarian cancer (OC) is the most lethal gynecologic malignancy among women. An estimate of 21,750 new cases of OC will be diagnosed, and 13,940 women will die of OC in the United States in 2020 according to the American Cancer Society (1). The standard treatment for advanced OC is primary cytoreductive surgery followed by adjuvant chemotherapy using a platinum analog plus paclitaxel (2–4). Poly ADP ribose polymerase inhibitors (PARPs) used in the management of epithelial ovarian cancer (EOC) have renewed the hope of patients with EOC. Unfortunately, the clinical effects of PARPi therapy in patients with OC are less favorable because of PARPi resistance (5–8). The prognosis for the advanced stage of OC remains poor even though a novel technology has been applied for the diagnosis and treatment of OC (9). We have gained insight into OC in recent years, although advancing knowledge has been previously hindered by substantial disease heterogeneity and uncertainties as to the origin of tumor tissues. Therefore, the identification of an ideal biological factor that predicts the malignant biological behavior and prognosis of OC will be helpful.

Liver receptor homolog 1 (LRH1), also known as nuclear receptor subfamily 5 group A member 2 NR5A2, participates in various biological processes, such as liver and pancreas differentiation, steroidogenesis in childhood, cholesterol/bile acid homeostasis, and tumor progression (10, 11). Previous studies validated that the overexpression of LRH1 may induce the resistance of breast cancer to chemotherapy (12, 13), the metastasis of pancreas cancer (14), the poor prognosis of colon cancer (15) and non-small cell lung cancer (16), and the proliferation of hepatoblastoma (17). However, LRH1 expression has not yet been reported in patients with OC.

Thus, we aimed to explore the status of LRH1 protein expression in OC and its clinical relevance. The functions of LRH1 were predicted using bioinformatics analysis.

## Materials and Methods

### Human Tissue Samples and Clinical Data

OC tumor tissue samples were obtained from Harbin Medical University Cancer Hospital between January 2009 and December 2011 from 133 patients with OC who underwent cytoreductive surgery without any prior treatment for cancer, such as chemotherapy or radiation. The International Federation of Gynecology and Obstetrics (FIGO) staging system was applied to assess tumor stage (18). Histological grades were based on the World Health Organization’s Histological Grading System for tumors (19). Furthermore, the subjects underwent lymph node dissection to assess lymph node status. Twenty-five patients with normal ovaries who underwent hysterectomy with oophorectomy in our hospital for benign uterine disease during the same period were also included.

Approval from the Medical Ethics Committee of Harbin Medical University Cancer Hospital was obtained for the purpose of research. The 133 patients with OC were followed up for survival analyses until death or until the study closing date (January 2018).

### Immunohistochemical Staining and Evaluation

133 tissue blocks of OC and 25 of normal ovary tissues were cut by a microtome into 4-µm sections and affixed onto the slide. After dewaxing in xylene and rehydrating through graded alcohol concentrations, the sections were incubated with 0.3% hydrogen peroxide for 10 min at room temperature to block endogenous peroxidase, and then the sections were incubated with anti-LRH1 antibody (1:100, Abcam, ab223211) overnight at 4°C. After washing in phosphate-buffered saline (PBS), all sections were incubated with secondary antibodies (R&D Systems, NL004) at room temperature for 20 min and then treated with 3,3′-diaminobenzidine tetrahydrochloride (Dako, Hamburg, Germany), followed by counterstaining with hematoxylin.

Protein expression level of LRH1 was scored by evaluating the percentage of the positive staining areas of tumor cells together with intensity of staining. The former was scored as follows (20): 0, < 0%; 1, 1–10%; 2, 11–50%; 3, 51–70% and 4, ≥70%. The latter was scored as follows: 0, negative staining; 1, weak staining; 2, moderate staining; and 3, intense staining. The final expression level of LRH1 was semi-quantitatively evaluated according to the sum of the scores for the percentage of positively stained tumor cells and intensity scores (0–7) in which the final staining scores of 0–3 and 4–7 were considered to be low and high expression, respectively.

The IHC staining on each slide was scored twice independently by two pathologists who were sophisticated in evaluating IHC and blinded to the clinicopathological information.

### Cell Culture

The human OC cell lines A2780CP, SKOV3 and OVCAR3 (ATCC) were employed. Both the cell lines have been approved by short tandem repeat (STR) profiling. Cells were cultured at 37°C in a humidified atmosphere containing 5% CO2 in DMEM complemented with 10% fetal bovine serum (FBS) (Hyclone, USA) and antibiotics (penicillin and streptomycin). Cells were passaged when they reached 80% confluence.

### Western Blotting Analysis

The cell lysates were both centrifuged at 13,000 rpm (at 4°C for 5 min). After that 10% sodium dodecyl sulfate polyacrylamide gel electrophoresis (SDS–PAGE) were used to segregate them and then transferred onto polyvinylidene difluoride (PVDF) membranes (Millipore, Bedford, MA, USA). The filters were blocked with blocking buffer (Sangon Biotech) for 45 min. Membranes were incubated with primary antibodies anti-LRH1 (Abcam, ab223211), anti-E-cadherin (Abcam, ab1416), anti-N-cadherin (Abcam, ab18203), anti-Vimentin (Abcam, ab92547), anti-*β*-catenin (Cell Signaling Technology, D10A8)) at 4°C overnight and with secondary antibodies at 1:5,000 dilution at room temperature for 2 h, followed by incubating with *β*-actin (Abcam, ab179467) at room temperature for 1.5 h.

### RNA Extraction and Reverse Transcription-qPCR

The TRIzol™ LS Reagent (Invitrogen™) was used to extract total RNA from tissues and cells, according to the manufacturer’s protocol followed by complementary DNA synthesis using a PrimeScript RT reagent Kit with gDNA Eraser (Takara). Amplification was subsequently carried out using the GeneAmp^®^ PCR System 9700 (Applied Biosystems, USA). Real-time PCR was performed using LightCycler^®^ 480 II Real-time PCR Instrument (Roche, Swiss). The mRNA expression levels of LRH-1 were normalized to the mRNA levels of GAPDH, which was used as an internal control. The 2^−ΔΔCt^ method was used to quantify the mRNA expression levels (21).

### Establishment of Stable Short Hairpin (sh)RNA-Mediated LRH-1 Knockdown Ovarian Cancer Cell Lines

shRNA-induced knockdown of LRH-1 expression was achieved using the lentiviral expression system. Human NR5A2 shRNA clone set was bought from GeneCopoeia (China). Viral particles were generated by co-transfecting 293T cells (ATCC) with the shRNAs and the Lenti-Pac™ Lentivirus Packaging kit (GeneCopoeia,China), which contains packaging plasmids and a transfection reagent according to the manufacturer’s protocol. Subsequently, the shRNA viral particles transfected SKOV3 and OVCAR3 cells with 4 μg/ml polybrene (Sigma-Aldrich), and stable cell lines were established after 10 days of puromycin (2 μg/ml) selection. Knockdown was confirmed using RT-qPCR or immunoblotting. The selected cell lines were routinely cultured in puromycin-containing media until 2 days prior to experimentation.

### Transwell Assay

For migration assays, infected OVCAR3 cells (1 × 10^5^ in 200 μl of serum-free DMEM medium) and SKOV3 cells (1 × 10^5^ in 200 μl of serum-free DMEM medium) were seeded into the upper chamber of transwell plates in a 24-well format with 8 μm diameters (Corning Costar, USA). Then, 600 μl of medium containing 10% FBS was added to the bottom chamber as a chemoattractant. After 24 h of culture, cells were fixed with methanol and stained with Crystal Violet solution. The remaining cells were removed from the top of the permeable membrane using a cotton swab. Then, cells that migrated through the upper chamber were counted in four random fields under a light microscope [NIKON INSTRUMENTS (SHANGHAI) CO., LTD].

### Wound Healing Assay

OVCAR3 (1 × 10^5^ cells) and SKOV3 cells (1 × 10^6^ cells) were seeded in 6-well cell culture plates and incubated for 24 h at 37°C. After achieving confluence, the cellular layer in each plate was scratched using a plastic pipette tip. The migration of the cells at the edge of the scratch was analyzed at 0 and 24 h when microscopic images of the cells were captured.

### Cell Counting Kit-8

SKOV3 and OVCAR3 cells were plated in flat-bottom 96-well plates (1,500 and 1,000 cells/well) and supplemented with 100 μl DMEM medium with 10% FBS per well. After incubation at 37°C in a humidified incubator with 5% CO2 for 2, 24, 48, 72, and 96 hours, respectively, 10 μl of CCK-8 (Yeasen Biotech Co., Ltd.) was added to each well. Then, after 1 h of culture, colorimetric analysis was performed on a microplate reader (Thermo Scientific™ Varioskan™ LUX) at a wavelength of 450 nm. The assay was performed using six replicates.

### Compounds and Drug Treatments

A2780CP-sh LRH1 and A2780CP-sh NC cells were seeded in 96-well culture plates, respectively; cells were treated with CDDP (Selleck) for 48 h at 37°C at the indicated concentrations. Cell viability was determined after treatment using the CCK-8 (Yeasen Biotech Co., Ltd.).

### Statistical Analysis

The chi-square test was used to analyze the statistical differences of the clinicopathologic variables. Overall survival (OS) and disease-free survival (DFS) were evaluated by the Kaplan–Meier method and log-rank test. The Cox proportional hazards model was used to estimate the independent prognostic factors for survival. Logistic regression was performed for multivariate analysis of the association between LRH1 expression and intraperitoneal metastasis. Student’s t-test was used to analyze the differences between the experimental and control groups. A two-sided P < 0.05 was considered significant.

### UALCAN

UALCAN (http://ualcan.path.uab.edu) is an interactive web resource based on TCGA database, which can be used to analyze relative transcriptional expression of potential genes of interest between tumor and normal samples and association of the transcriptional expression with relative clinicopathologic parameters (22). In this study, we use UALCAN to explore the association of LRH1 mRNA expressions in ovarian cancer tissues and clinicopathologic parameters. Student’s t test was applied to compare the difference of mRNA expression, and p < 0.01 was considered as statistically significant.

### GEPIA

GEPIA (http://gepia.cancer-pku.cn/) is a web server to analyze cancer and normal gene expression and interaction (23). In this study, we use GEPIA to obtain a series of genes that have similar expression patterns with LRH1 in ovarian cancer.

### Metascape

The Metascape (http://metascape.org/) is an online analytical tool, which facilitates gene annotation integration, functional enrichment, interactome analysis (24). In the present study, Metascape was applied for gene-enrichment analysis of genes that have similar expression patterns with LRH1 in ovarian cancer.

## Results

### Expression Level of LRH1 in OC Tissues

**Figure 1:**
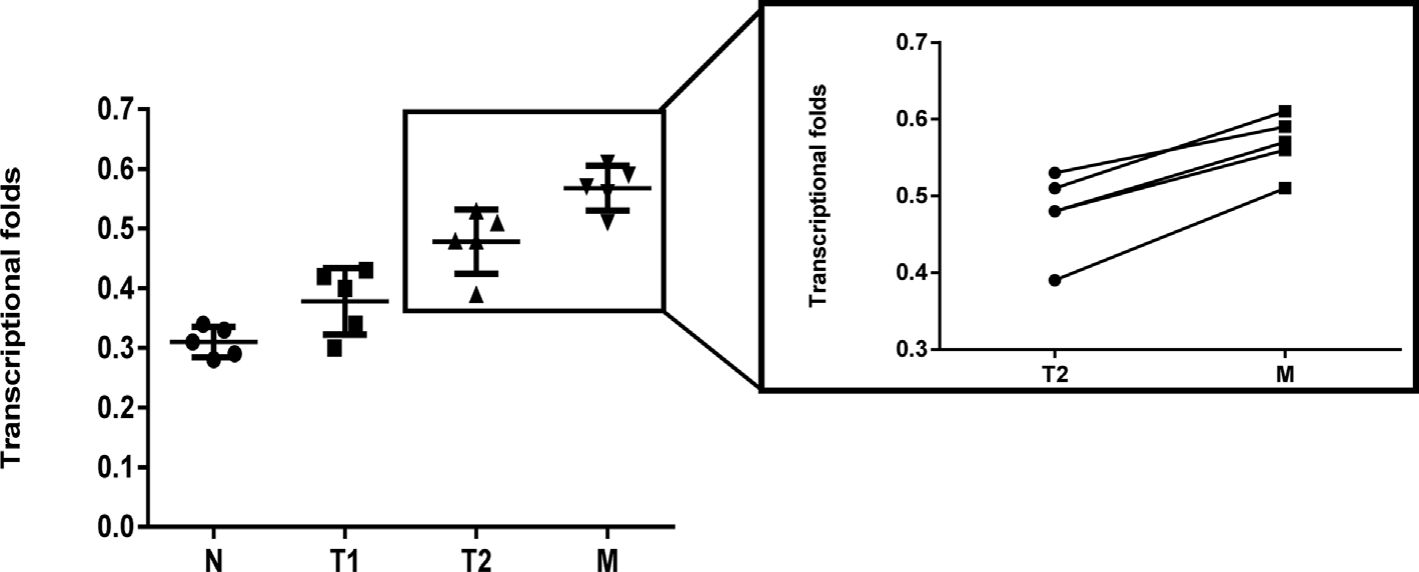
Real time PCR assay of LRH1 mRNA expression in N, T1, T2, and its matched M. It showed that elevated trend of LRH1 mRNA expression from N to T1, T2, and its matched M (*P* < 0.05).

RT-qPCR was carried out to determine the difference in the LRH1 mRNA expression between OC and normal ovarian tissues (**Figure 1**). No discrepancy was observed in the LRH1 mRNA expression between normal tissues and OC tissues without metastasis (T1) (*P* > 0.05). However, the LRH1 mRNA expression in OC tissues with metastasis present (T2) was higher than that in T1 (*P* = 0.017). Moreover, LRH1 mRNA expression was more intense in the invasive foci (M) than in the *in situ* foci (T2) among the same patients (*P* = 0.034).

**Figure 2:**
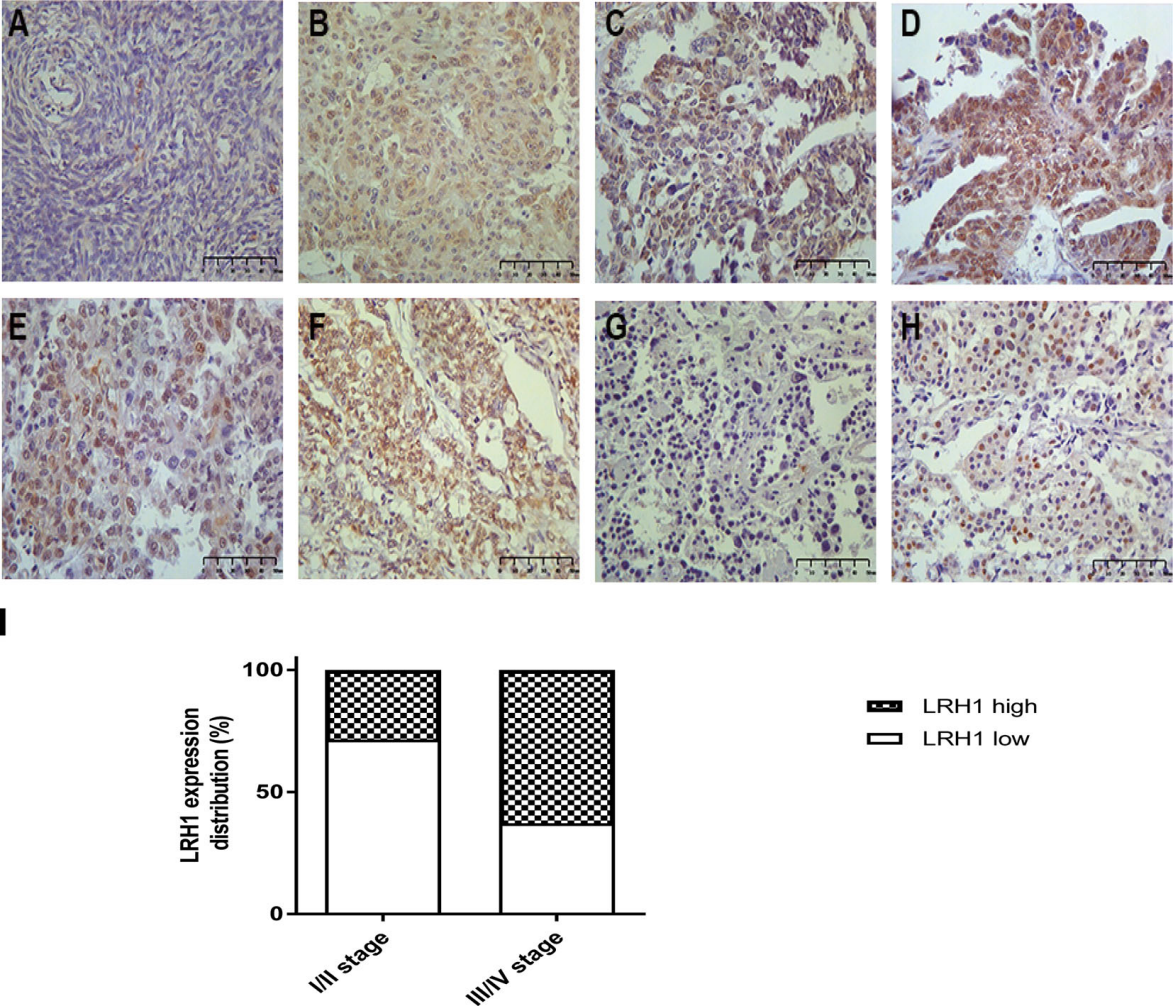
Immunohistochemical analysis of LRH1 protein expression (×400). **(A)** negative or weak expression in normal ovarian tissues; **(B)** high expression in primary ovarian cancer (OC) tissues (observed mainly in nucleus, a few in cytoplasmic); **(C)** low expression in serous histotypes (mainly in nucleus); **(D)** high expression in serous histotypes (mainly in nucleus, a few in cytoplasmic); **(E)** high expression in mucinous histotypes (mainly in nucleus); **(F)** high expression in endometrioid histotypes (mainly in nucleus, a few in cytoplasmic); **(G)** negative or low expression in clear cell histotypes; **(H)** high expression in clear cell histotypes (mainly in nucleus, scarcely in cytoplasmic); **(I)** the status of LRH1 expression in different FIGO stages.

IHC revealed that the expression of LRH1 was scarcely detectable in normal tissues but obvious in OC tissues (**Figure 2**). As shown in **Figure 2B**, LRH1 was observed in the nucleus and cytoplasm of tumor cells but was localized in the cytoplasm of cells in normal tissues. Among the 133 OC specimens examined, 59 (44.4%) had low LRH1 expression and 74 (55.6%) had high LRH1 expression. Furthermore, the patients with OC who had high LRH1 expression comprised 28.6% of patients with FIGO I/II and 62.9% of patients with FIGO III/IV.

LRH1 mRNA expression patterns were further measured by UALCAN. The data resources of UALCAN were based on The Cancer Genome Atlas database. As shown in **Figure 4A**, LRH1 mRNA expression was upregulated in stage II and stage IV OC tissues compared with stage I and stage III OC tissues, but the difference was not statistically significant.

### Relationship of Elevated LRH1 Protein Expression With Clinicopathologic Variables

**Table 1** summarizes the association between LRH1 expression and clinicopathological variables in OCs. LRH1 expression was significantly related to FIGO stage (*P* = 0.001), lymph node metastasis (*P* = 0.011), intraperitoneal metastasis (*P* = 0.001), and platinum resistance (*P* = 0.037). LRH1 expression was not related to age, histological type, histologic grade, residual disease, ascites, and serum CA-125 level (*P* > 0.05).

**Table 1:** Correlation between LRH1 protein expression level and clinicopathological variables in 133 patients with OC.

Variables	All patientsn = 133	LRH1 expression level		*P*
		Low (%) (n = 59)	High (%) (n = 74)	
Age (years)				0.252
≤52	67	33 (49.3)	34 (50.7)	
>52	66	26 (39.4)	40 (60.6)	
Histological type Serous	92	42 (45.7)	50 (54.3)	0.653
Others	41	17 (41.5)	24 (58.5)	
FIGO stage				0.001
I/II	28	20 (71.4)	8 (28.6)	
III/IV	105	39 (37.1)	66 (62.9)	
Histologic grade				0.310
G1/G2	50	25 (50.0)	25 (50.0)	
G3	83	34 (41.0)	49 (59.0)	
Residual disease (cm)				0.851
<1	98	43 (43.9)	55 (56.1)	
≥1	35	16 (45.7)	19 (54.3)	
Ascites (ml)				0.473
<100	38	15 (39.5)	23 (60.5)	
≥100	95	44 (46.3)	51 (53.7)	
Serum CA-125 level (U/ml)				0.653
≤35	41	17 (41.5)	24 (58.5)	
>35	92	42 (45.7)	50 (54.3)	
Lymph node metastasis				0.011
No	81	43 (53.1)	38 (46.9)	
Yes	52	16 (30.8)	36 (69.2)	
Peritoneum metastasis				0.001
No	43	28 (65.1)	15 (34.9)	
Yes	90	31 (34.4)	59 (65.6)	
Platinum resistance				0.037
No	67	33 (49.3)	30 (50.7)	
Yes	66	26 (39.4)	44 (60.6)	

FIGO, the Federation of Gynaecology and Obstetrics; G1, Well differentiated; G2, moderately differentiated; G3, poorly differentiated; OC, ovarian cancer.

### Increased LRH1 Expression in OC Predicts Unfavorable Prognosis and Recurrence

**Figure 3:**
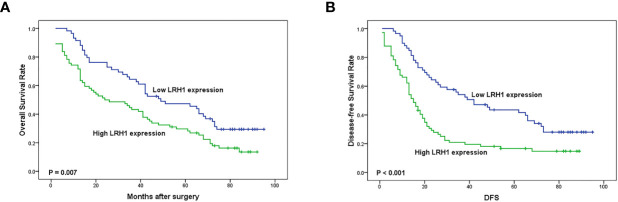
Kaplan–Meier analysis of overall survival (OS) and disease-free survival (DFS) related to the expression of LRH1. **(A)** OS curves of the 133 OC patients; **(B)** DFS curves of the 133 OC patients.

Log-rank test showed that patients with low LRH1 expression had a significantly better OS (*P* = 0.007) and DFS (*P* = 0.001) compared with patients with high LRH1 (**Figure 3**). FIGO stage (both *P* < 0.001), histologic grade (*P* = 0.009 0.028, respectively), residual disease (*P* = 0.022 and 0.026, respectively), lymph node metastasis (both *P* < 0.001), and intraperitoneal metastasis (*P* = 0.001 0.001, respectively) were correlated with the prognosis of patients with OC regardless of low and high LRH1 expression (**Table 2**). Multivariate Cox regression analysis showed that high LRH1 expression was considered an independent prognostic marker for the DFS of patients with OC instead of OS (*P* = 0.041; **Table 2**).

**Table 2:** Univariate and multivariate analyses for survival in 133 OC patients.

Variables	Univariate		Multivariate	
	*P*	HR	95%CI	*P*
variables related to OS			
Age (years)	0.219			
Histological type	0.914			
FIGO stage	<0.001	3.695	(1.793–7.613)	<0.001
Histologic grade	0.009	1.664	(1.101–2.513)	0.016
Residual disease (cm)	0.022	1.682	(1.093–2.591)	0.018
Ascites (ml)	0.696			
Serum CA-125 level (U/ml)	0.154			
Lymph node metastasis	<0.001	3.665	(2.344–5.730)	<0.001
Peritoneum metastasis	0.001			
LRH1 expression level	0.007			
variables related to DFS			
Age (years)	0.487			
Histological type	0.895			
FIGO stage	<0.001	3.437	(1.641–7.200)	0.001
Histologic grade	0.028	1.550	(1.024–2.345)	0.038
Residual disease (cm)	0.026	1.663	(1.065–2.597)	0.025
Ascites (ml)	0.855			
Serum CA-125 level (U/ml)	0.281			
Lymph node metastasis	<0.001	3.857	(2.431–6.120)	<0.001
Peritoneum metastasis	<0.001			
LRH1 expression level	<0.001	1.535	(1.017–2.318)	0.041

FIGO, the Federation of Gynaecology and Obstetrics; OS, overall survival; DFS, disease-free survival; OC, ovarian cancer; HR, hazard ratio; CI, confidence interval.

### Increased LRH1 Expression as Indicator of Intraperitoneal and Lymph Node Metastases in OC

The traditional clinicopathological parameters of intraperitoneal metastasis were first appraised to evaluate the relationship between LRH1 overexpression and intraperitoneal metastasis (**Table 3**). Univariate analyses of clinicopathologic variables for intraperitoneal metastasis showed that the presence of intraperitoneal metastasis was positively associated with FIGO stage (*P* < 0.001), residual disease (*P* = 0.025), and ascites (*P* = 0.019). Multivariate logistic regression analysis revealed that FIGO stage (*P* = 0.003), residual disease (*P* = 0.032), and LRH1 (*P* = 0.010) were independently linked with intraperitoneal metastasis.

**Table 3:** Univariate and multivariable analyses for the relationship between LRH1 expression and lymph node metastasis in OCs.

Variables	No.	Lymph node metastasis		P	OR (95%CI)	P
		Negative	Positive			
Age (years)				0.775		
≤52		40	27			
>52		41	25			
Histological type				0.991		
Serous		56	36			
Others		25	16			
Histologic grade				0.016	2.421 (1.109–5.285)	0.026
G1/G2		37	13			
G3		44	39			
Residual disease (cm)				0.595		
<1		61	37			
≥1		20	15			
Ascites (ml)				0.653		
<100		22	16			
≥100		59	36			
Serum CA-125 level (U/ml)				0.991		
≤35		25	16			
>35		56	36			
LRH1 expression level				0.011	2.451 (1.162–5.173)	0.019
Low		43	16			
High		38	36			

OC, ovarian cancer; HR, hazard ratio; CI, confidence interval.

Results showed that the presence of lymph node metastasis was significantly correlated with histologic grade (*P* = 0.016) and LRH1 expression (*P* = 0.011). Multivariate logistic regression showed that histologic grade (*P* = 0.026) and LRH1 expression (*P* \= 0.019) significantly led to lymph node metastasis (**Table 4**).

**Table 4:** Univariate and multivariable analyses for the relationship between LRH1 expression and peritoneum metastasis in OCs.

Variables	No.	Peritoneum metastasis		P	OR (95%CI)	P
		Negative	Positive			
Age (years)				0.900		
≤52		22	45			
>52		21	45			
Histological type				0.918		
Serous		30	62			
Others		13	28			
FIGO stage				<0.001	4.613 (1.678–12.677)	0.003
I/II		19	9			
III/IV		24	81			
Histologic grade						
G1/G2		21	29	0.064		
G3		22	61			
Residual disease (cm)					3.208 (1.104–9.322)	0.032
<1		37	61	0.025		
≥1		6	29			
Ascites (ml)						
<100		18	20	0.019		
≥100		25	70			
Serum CA-125 level (U/ml)						
≤35		12	29	0.614		
>35		31	61			
LRH1 expression level					3.160 (1.323–7.545)	0.010
Low		28	31	0.001		
High		15	59			

FIGO, the Federation of Gynaecology and Obstetrics; OC, ovarian cancer; HR, hazard ratio; CI, confidence interval.

### LRH1 Knockdown Inhibits FOC Cell Proliferation and Migration In Vitro

**Figure 4:**
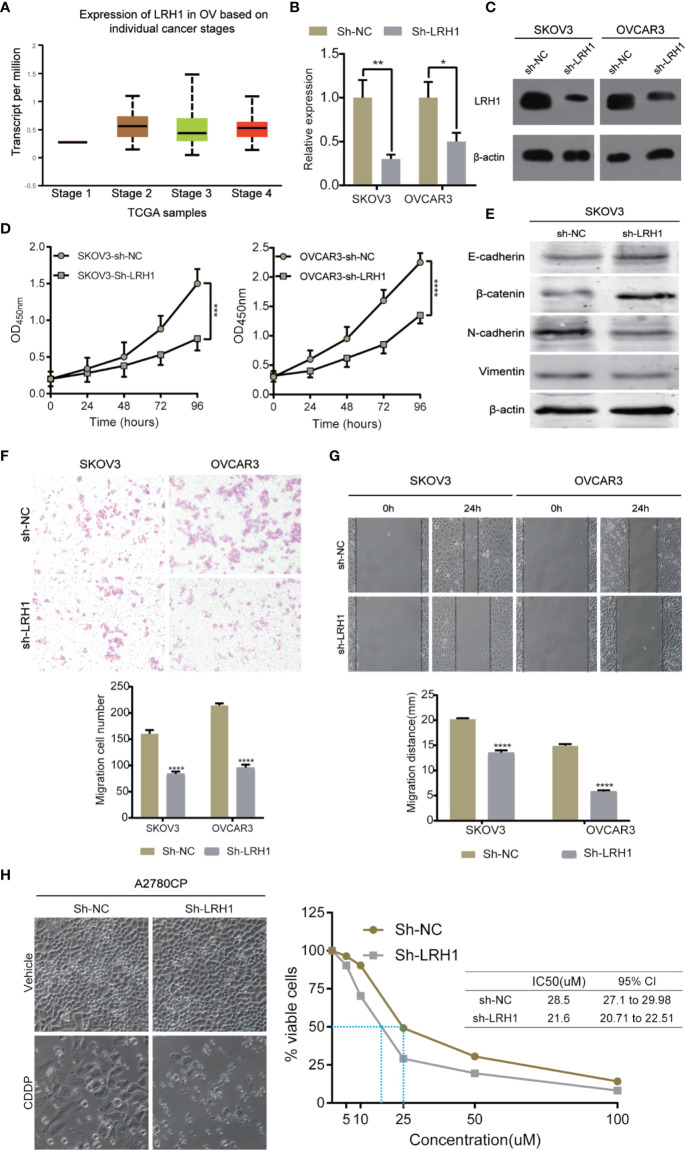
Knockdown of LRH1 inhibits ovarian cancer cell proliferation and migration *in vitro*. **(A)** The mRNA expression of LRH1 in ovarian cancer (UALCAN). The level of mRNA **(B)** and protein **(C)** expression significantly decreased after sh-LRH1 transfection. CCK8 assays **(D)** have revealed that knockdown of LRH1 inhibits cell proliferation. **(E)** Western blotting was applied to detect E-cadherin, *β*-catenin, N-cadherin, vimentin, and *β*-actin with the respective antibodies. Transwell assays **(F)** and wound healing assays **(G)** have indicated that knockdown of LRH1 inhibits cell migration. **(H)** LRH1 knockdown enhanced sensitivity to cisplatin in A2780CP cells. ^****^P < 0.0001,^***^P < 0.001, ^**^P < 0.01, ^*^P < 0.05; data are shown as the mean ± SD.

LRH1 knockdown assays using sh-RNA was conducted to investigate the biological function of LRH1 in OC cells. We used RT-qPCR and Western blotting to confirm that the LRH1 expression in OVCAR3 and SKOV3 cells transfected with sh-LRH1 was remarkably lower than in those transfected with sh-NC (**Figures 4B, C**). We explored the effect of LRH1 knockdown on the proliferation of OVCAR3 and SKOV3 cells. The proliferation of OVCAR3 and SKOV3 cells infected with sh-LRH1 was repressed at 24, 48, 72, and 96 h, respectively, compared with OVCAR3 and SKOV3 cells infected with sh-NC (**Figure 4D**). Thus, LRH1 knockdown substantially repressed cell proliferation.

We also investigated the effect of LRH1 on cell migration. Epithelial–mesenchymal transition (EMT) plays a crucial role in tumor invasion and metastasis in OC. We used Western blot to detect the protein levels of EMT markers, namely E-cadherin, N-cadherin, vimentin, and *β*-catenin. The results showed that E-cadherin and *β*-catenin level increased in LRH1 knockdown cells, whereas N-cadherin and vimentin decreased. Moreover, transwell assay was conducted to evaluate cell migration. Migrated cells were counted in three random fields in transwell plates, and the average value of the three fields was calculated. The result of the transwell assay showed that knockdown of LRH1 decreased the migration of SKOV3 and OVCAR3 cells by approximately 42 and 50%, respectively (**Figure 4F**). Wound-healing assay showed that LRH1 promoted the migration of SKOV3 and OVCAR3 cells **(****Figure 4G**). After 24 h, control SKOV3 and OVCAR3 cells migrated approximately 20 and 15 mm, respectively, whereas SKOV3 and OVCAR3 cells with LRH1 knockdown migrated approximately 13 and 6 mm, respectively. The cells with LRH1 knockdown showed less motility than control cells; thus, LRH1 could stimulate cell migration.

Moreover, we conducted CCK8 assay to evaluate whether LRH1 could mediate the effects of cell survival after CDDP treatment. Results showed that knockdown of LRH1 decreased CDDP half-maximal inhibitory concentration (IC50) in A2780CP cells (**Figure 4H**).

### Functional Enrichment Analysis of LRH1 in OC

**Figure 5:**
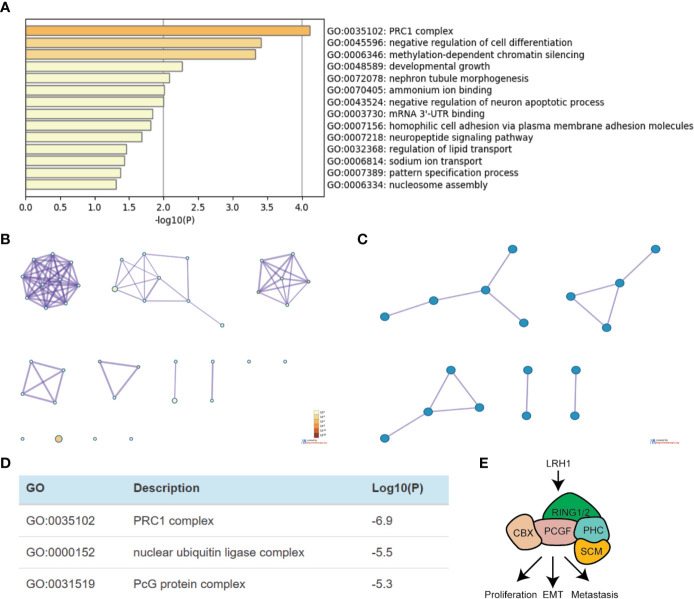
Functional enrichment analysis of genes that have similar expression pattern to LRH1 in ovarian cancer (Metascape). **(A)** Heatmap of Gene Ontology (GO) and enriched terms colored by p-values. **(B)** Network of GO enriched terms colored by p-value. **(C)** Protein–protein interaction network and MCODE components identified in the gene lists. **(D)** Independent functional enrichment analysis of MCODE components. **(E)** A schematic model depicting LRH1 upregulation probably promotes cell proliferation, EMT, metastasis by activating PRC1 complex in ovarian cancer cells *via* bioinformation analysis.

We obtained 230 genes that have the same expression pattern as LRH1 through Gene Expression Profiling Interactive Analysis as described in the *Materials and Method* section to evaluate the function of LRH1 in OC. We aimed to explain the mechanisms of LRH1 OC to determine the biological functions of these similar genes. We uploaded the 230 similar genes into Metascape and conducted custom analysis. As shown in **Figures 5A, B**, the function of these similar genes was related to the polycomb repressive complex 1 (PRC1 complex), the negative regulation of cell differentiation, methylation-dependent chromatin silencing, developmental growth, nephron tubule morphogenesis, ammonium ion binding, the negative regulation of neuron apoptotic process, mRNA mRNA 3′ untranslated region (UTR) binding, homophilic cell adhesion *via* plasma membrane adhesion molecules, neuropeptide signaling pathway, the regulation of lipid transport, sodium ion transport, pattern specification process, and nucleosome assembly. Moreover, we applied Metascape to explore the protein–protein interactions of the genes similar to LRH1. As shown in **Figures 5C, D**, the protein–protein interaction network was related to the PRC1 complex, nuclear ubiquitin ligase complex, and polycomb group (PcG) proteins.

## Discussion

OC is the most lethal cancer among women, and its 5-year survival rate in advanced stages is 30–40% (25). The American Cancer Society reported that about 21,750 new cases of OC will be diagnosed, and 13,940 women will die of OC in the United States in 2020 (1). OC is a heterogeneous tumor that contains a heterogenous group of malignancies, which differ in etiology, molecular biology, and other characteristics (26). High-risk OC has very low cure rates despite therapy that comprised surgery, chemotherapy, PARPi, immunotherapy, and radiation (26). Clearly, the identification of novel biomarkers and more effective therapies is extremely urgent.

LRH1 is a member of the nuclear receptor NR5A (Ftz-F1) subfamily, which is expressed in the endodermal tissues of the intestine, liver, exocrine pancreas (27), ovary (28–31), pre-adipocyte (32), and placenta (33). Moreover, LRH1 plays an important role in a variety of biological functions, including cell proliferation, differentiation, and tumorigenesis.

In the present study, we applied RT-qPCR to detect the mRNA level of LRH1 in patients with OC. Results showed the increasing trend of LRH1 mRNA expression from N to T1, T2, and its matched M (**Figure 1**). This may indicate that the more OC tissue expresses LRH1 the more malignant potential it has. Moreover, we conducted immunohistochemical (IHC) assays to detect the protein level of LRH1. IHC revealed that LRH1 protein expression in normal tissues was scarcely detectable in normal tissues but intense in OC tissues (**Figure 2**). We also explored the relationship between LRH1 protein expression and clinicopathological factors and found that LRH1 predicts peritoneal metastasis and poor outcome. Therefore, LRH1 has a potential role as a biomarker of OC (**Tables 1**–**4**, **Figure 3**). Furthermore, the mRNA expression level of LRH1 was confirmed by UALCAN. **Figure 4A** shows that LRH1 mRNA expression was upregulated OC tissues in stages II and IV compared with those in stages I and III, but the difference was not statistically significant. *In vitro* assays showed that LRH1 could promote tumor cell proliferation, migration, and EMT (**Figures 4D–G**). In addition, it also could promote cisplatin resistance (**Figure 4H**). Bioinformatics analysis was applied to investigate the biological function of LRH. Results demonstrated that the functions of LRH1 are associated with the PRC1 complex, the negative regulation of cell differentiation, methylation-dependent chromatin silencing, developmental growth, nephron tubule morphogenesis, ammonium ion binding, negative regulation of neuron apoptotic process, mRNA 3′ UTR binding, homophilic cell adhesion *via* plasma membrane adhesion molecules, neuropeptide signaling pathway, lipid transport regulation, sodium ion transport, pattern specification process, and nucleosome assembly (**Figure 5**).

The protein and mRNA levels of LRH1 are upregulated in various cancers (15–17). We found the same conclusion in our results. Lin et al. viewed LRH1 as a remarkable prognosis biomarker for tumor invasion and proliferation in pancreas cancer (34). Thus, we were prompted to identify the role of LRH1 overexpression as a predictor of clinicopathological and prognostic relevance. We recognized that elevated LRH1 protein expression was strongly associated with FIGO stage, lymph node metastasis, and intraperitoneal metastasis. Furthermore, multivariate logistic regression model demonstrated that high LRH1 expression was remarkably associated with lymph node and intraperitoneal metastases. In addition, the OS periods of patients with high LRH1 expression were shorter than those of patients with low LRH1 expression. The patients with low LRH1 expression presented better prognoses than those with high LRH1 expression. The *in vitro* assay of OC cells showed that LRH1 could promote tumor cell proliferation, migration, and EMT. Our findings are consistent with the results of previous research.

LRH1 protein functions as a cancer regulator that exists in extensive procedures, such as cell proliferation, chemotherapy resistance, and tumor progression (12, 13, 35). The mechanism of LRH1 that influences migration demonstrated that LRH1 enhanced the transcriptional activity of *β*-catenin and upregulated the expression of downstream target genes (c-Myc and MMP2/9); thus, LRH1 promoted cell migration and invasion (14). Furthermore, we found that LRH1 promoted cisplatin resistance. Similar with the above, LRH1-induced chemotherapy resistance was reported in breast cancer. LRH1 promotes the chemoresistance of breast cancer cells by enhancing the expression of MDC1 and attenuating DNA damage (13). Bianco et al. found that LRH1 could increase the level of cyclin-dependent kinase inhibitor CDKN1A in a p53-independent manner, which results in tumor cell proliferation (36). Bioinformatics analysis was applied to evaluate the function of LRH1 in OC and demonstrated that the functions of LRH1 are associated with the PRC1 complex, the negative regulation of cell differentiation, methylation-dependent chromatin silencing, developmental growth, nephron tubule morphogenesis, ammonium ion binding, the negative regulation of neuron apoptotic process, mRNA 3′ UTR binding, homophilic cell adhesion *via* plasma membrane adhesion molecules, neuropeptide signaling pathway, lipid transport regulation, sodium ion transport, pattern specification process, and nucleosome assembly. These findings suggest the important biological role of LRH1 in carcinogenesis and tumor progression.

## Conclusion

Although limitations still exist in this study, the results we observed showed that LRH1 contributes to lymph node metastasis, intraperitoneal metastasis, and poor prognosis in OC. Thus, LRH1 could be a specific biomarker for predicting poor prognosis and metastasis. Furthermore, LRH1 promotes tumor cell proliferation, migration, EMT, and cisplatin resistance *in vitro*. Bioinformatics analysis predicted that the functions of LRH1 are associated with PRC1 complex, nuclear ubiquitin ligase complex, and PcG proteins.

## Data Availability Statement

The raw data supporting the conclusions of this article will be made available by the authors, without undue reservation.

## Ethics Statement

The studies involving human participants were reviewed and approved by the Ethics Committee of Harbin Medical University Cancer Hospital. The patients/participants provided their written informed consent to participate in this study.

## Author Contributions

LY conceived and designed the study. JXL and JML collected samples and processed the data. QS provided technical support. WS and LY analyzed the data. WS drafted the manuscript. LY revised the manuscript. All authors contributed to the article and approved the submitted version.

## Funding

This study was supported by the Science and Technology Project of Education Department of Heilongjiang Province (12531275).

## Conflict of Interest

The authors declare that the research was conducted in the absence of any commercial or financial relationships that could be construed as a potential conflict of interest.

## Abbreviations

LRH1, liver receptor homolog 1; OC, ovarian cancer; RT, reverse transcription; FIGO, the International Federation of Gynecology and Obstetrics; PBS, phosphate-buffered saline; IHC, Immunohistochemical; STR, short tandem repeat; SDS–PAGE, sodium dodecyl sulfate polyacrylamide gel electrophoresis; PVDF, polyvinylidene difluoride; OS, Overall survival; DFS, disease-free survival; EMT, epithelial–mesenchymal transition; PRC1 complex, polycomb repressive complex 1; UTR, untranslated region.
